# DESIGN ELEMENTS OF EDUCATIONAL TOYS FOR PATIENTS WITH MILD ALZHEIMER’S DISEASE: A QUALITATIVE STUDY

**DOI:** 10.1093/geroni/igae098.3578

**Published:** 2024-12-31

**Authors:** HongFei Jia, YaPing Ding, Ling Zhu

**Affiliations:** Nanjing Medical University School of Nursing, Nanjing City, Jiangsu, China (People’s Republic); Nanjing Medical University School of Nursing, Nanjing City, Jiangsu, China (People’s Republic); Nanjing Medical University School of Nursing, Nanjing City, Jiangsu, China (People’s Republic)

## Abstract

**Background:**

Alzheimer’s disease (AD) is a fatal chronic neurodegenerative disease, and its incidence and mortality rate are closely related to age. Once diagnosed, patients need to take long-term medication, which seriously affects the quality of life of those who are ill. Research has demonstrated that the educational toy is an effective intervention to improve cognitive function among patients with mild Alzheimer’s disease. Despite the rapid growth in developing educational toys, incorporation of stakeholders’ views has been limited. Objective: This project aimed to identify the design elements that should be considered during development of educational toys for patients, from the perspectives of patients with mild Alzheimer’s disease and their caregivers.

**Materials and Methods:**

A total of 10 patients with mild Alzheimer’s disease and 12 caregivers were selected. Participants were recruited using purposive sampling. Semi-structured interviews were conducted between August and November 2023. The content analysis method was used to summarize the interview data and refine the themes.

**Results:**

Main themes that emerged from the data analysis included the following: (1) materials; (2) structure; (3) function; (4) color; (5) shape.

**Conclusion:**

Collecting patients’ and caregivers’ perspectives through qualitative interviews, the developed puzzle toy can serve as a user-friendly and engaging tool to potentially complement traditional interventions in slowing cognitive deterioration in patients with mild Alzheimer’s disease. These design elements can also be considered by developers and researchers when developing and evaluating educational toys for people with mild Alzheimer’s disease.

